# A National Survey of Teachers on Antiretroviral Therapy in Malawi: Access, Retention in Therapy and Survival

**DOI:** 10.1371/journal.pone.0000620

**Published:** 2007-07-18

**Authors:** Simon D. Makombe, Andreas Jahn, Hannock Tweya, Stuart Chuka, Joseph Kwong-Leung Yu, Mindy Hochgesang, John Aberle-Grasse, Lameck Thambo, Erik J. Schouten, Kelita Kamoto, Anthony D. Harries

**Affiliations:** 1 Clinical HIV Unit, Ministry of Health, Lilongwe, Malawi; 2 Lighthouse Trust, Lilongwe, Malawi; 3 International Training and Education Center on HIV, Seattle, United States of America; 4 Malawi Business Coalition against AIDS, Blantyre, Malawi; 5 Taiwan Medical Mission, Mzuzu Central Hospital, Mzuzu, Malawi; 6 Global AIDS Program, United States Centres for Disease Control and Prevention, Malawi; 7 Management Sciences for Health, Lilongwe, Malawi; 8 Family Health International, Malawi Country Office, Lilongwe, Malawi; 9 London School of Hygiene and Tropical Medicine, London, United Kingdom; National AIDS Research Institute, India

## Abstract

**Background:**

HIV/AIDS is having a devastating effect on the education sector in sub-Saharan Africa. A national survey was conducted in all public sector and private sector facilities in Malawi providing antiretroviral therapy (ART) to determine the uptake of ART by teachers and their outcomes while on treatment.

**Methodology/Principal Findings:**

A retrospective cohort study was carried out based on patient follow-up records from ART Registers and treatment master cards in all 138 ART clinics in Malawi; observations were censored on September 30^th^ 2006. By this date, Malawi's 102 public sector and 36 private sector ART clinics had registered a total of 72,328 patients for treatment. Of these, 2,643 (3.7%) were teachers. Adjusting for double-registration caused by clinic transfers, it is estimated that 2,380 individual teachers had ever accessed ART. There were 15% of teachers starting ART in WHO clinical stage 1 or 2 with a CD4-lymphocyte count of ≤250/mm^3^ and 85% starting in stage 3 or 4. By 30^th^ September 2006, 1,850 teachers were alive on ART (3.5% of all teachers in Malawi). The probability of being alive on ART at 6-months, 12-months, 18-months and 24-months after treatment initiation was 84%, 79%, 75% and 73% respectively. Retention in treatment was better for women (adjusted HR = 1.8) and in those starting ART in WHO Clinical Stage 1 and 2 (adjusted HR = 1.8).

**Conclusion/Significance:**

Rapid scale up of ART has allowed 2,380 HIV-positive teachers to access life-prolonging treatment. There is evidence that this intervention can help to mitigate some of the shortages of teaching personnel in resource-poor countries affected by a generalised HIV epidemic.

## INTRODUCTION

The dual campaigns of Education for All (EFA) and the Millennium Development Goals (MDG) have made universal primary school enrolment for boys and girls a priority for developing countries [1]. The HIV and AIDS epidemic in many of these countries, however, is placing enormous challenges on the education sector to attain these goals. Sub-Saharan Africa is already facing a serious teacher shortage and AIDS-related sickness and mortality is exacerbating this problem [2]. HIV and AIDS decrease the supply and quality of education through increased absenteeism and mortality, resulting in loss of experienced teachers and increased class sizes. AIDS mortality also substantially increases the education wage bill, as attrition costs are high and death benefits soar.

Malawi, a small poverty stricken country in Southern Africa, faces a severe HIV epidemic. A study of the impact of AIDS on the public sector in Malawi found that HIV-related annual attrition in the education sector was 1.2% between 1990 and 2000, with HIV-related deaths responsible for at least 50% of the attrition [3]. By 1997, over 10% of education personnel in urban areas of Malawi were estimated to have died from AIDS with figures projected to rise to about 40% by 2005 [4]. A survey of primary school teachers in semi-urban and rural areas of the country in 1999 found an annual death rate of 2.3%, largely as a result of AIDS and TB [5]. Although representative empirical data are not available, HIV is likely to have severely aggravated the crisis in the education sector in Malawi.

Along with many other countries in sub-Saharan Africa, Malawi is scaling up antiretroviral therapy (ART) [6]. Data on all ART patients are recorded using a standardised system for monitoring of access and treatment outcomes, and this includes information on occupation. The system is rigorously supervised and the data can be considered nationally complete, offering the opportunity to examine access, retention in therapy and survival for subgroups of the population.

We have found no published data on teachers accessing ART in sub-Saharan Africa that would allow assessing the impact of free ART scale up on the education sector. We therefore conducted an audit using routinely collected data from Malawi's national monitoring and evaluation (M&E) system for ART.

## METHODS

### Background:

ART became available in Malawi in 2000 at central hospitals and a few non-governmental organizations. At this time, there were no national guidelines and different treatment regimens were typically offered on a subsidised cost basis. When the national roll-out program of free ART started in June 2004 an estimated 6,000 patients had received ART from 10 ART clinics in the country (source: HIV Unit, Ministry of Health, Malawi).

To facilitate rapid scale-up in the context of Malawi's resource-constrained public health system, the national program focused on a simple, standardised approach, which has been previously described [7]: use of one generic, fixed dose combination treatment (stavudine, lamivudine, nevirapine); a standardised system of registration, monitoring and reporting of cases and outcomes; and quarterly monitoring and supervision visits to all ART sites. ART-eligibility is routinely determined by clinical staging (WHO Clinical stage 3 or 4); patients in Clinical Stage 1 or 2 are eligible if their CD4-lymphocyte count is ≤250/mm^3^. However, CD4-lymphocyte counts are only available at a few specialised facilities. Patients are seen at two weeks after ART initiation and then routinely every month for clinical assessment and ART-dispensing. The visit interval is extended to 2 months if patients have completed 6 months of ART without complications. Patients with drug side effects are referred to experienced specialised sites where alternative ART regimens can be initiated. The process of ART scale up in the private sector has followed a similar approach using an identical monitoring system; treatment is offered here at subsidised cost (approximately USD$ 3.5 per course of treatment per month).

Malawi's national monitoring system for ART uses one patient master card for each patient and one ART register per facility [7]. At enrolment, patient demographics, occupation, stage defining conditions and clinical stage are recorded on the master card and copied into the register. At every ART visit, follow-up details are entered in the master card, including ambulatory and working status, pill-count, ART-regimen and drug side-effects. Follow-up outcomes such as transfer to another ART clinic, treatment discontinuation and death are also entered in the master card. A patient cohort analysis is conducted at all sites every quarter [8]. In preparation for this, clinic staff systematically review the follow-up status of all patients, updating the master cards and register with the latest outcomes. Patients who have failed to return for 3 months are marked as ‘defaulters’. Active follow-up of ‘defaulters’ has not been made mandatory due to resource constrains. However, more than half of the facilities in the national programme are consistently attempting to trace defaulters through community visits.

The HIV Unit of the Ministry of Health and its partners conduct quarterly supervision and monitoring visits to all ART sites in the country [8]. The supervisors check the accuracy, completeness and consistency of the register and master cards. Cohort analyses are checked and collected for aggregation and national level reporting.

### Data Collection and Analysis

The survey was conducted during supervision visits to all 102 public sector and 36 private sector ART clinics, which took place between October and December 2006. All ART clinic registers were screened for teachers who had accessed ART up to September 30^th^ 2006. For all teachers identified, the following data were transcribed onto a structured form: Site-specific registration number; sex; date, age and WHO clinical stage at ART initiation; pulmonary or extra-pulmonary TB, Kaposi's sarcoma as a stage-defining condition; date and type of follow-up outcome.

Data were checked, entered and cleaned in MS Access and analysed using STATA 9.2. For the survival analysis, teachers were considered to come under observation on the date of ART initiation if this was after June 1^st^ 2004, when the standard monitoring system in the national free ART program had been established. Teachers who had accessed ART before that date were considered to come under observation in June 2004, but were entered into the analysis taking account of the accumulated time on ART. Due to incomplete active ascertainment of deaths in the national monitoring and evaluation system, separate analyses were performed for deaths alone (treating loss to follow-up, ART discontinuation and transfers to other ART clinics as censoring events), and ‘ART drop-outs’ (combining deaths, loss to follow-up and ART discontinuation as ‘failure events’ and transfers to other ART clinics as censoring events). Observations were censored if patients were alive and on ART by 30^th^ September 2006. The probability of survival on ART was estimated using the Kaplan-Meier method. The effect of background characteristics on survival was measured using hazard ratios, and Poisson regression models were used for multivariate analyses.

### Ethical approval

General measures are provided in all ART facilities to ensure patient confidentiality, consent for HIV testing, and counselling and support for those who receive a positive HIV test result. Data collected for this study did not include personal identifiers. The Malawi National Health Science Research Committee provides general oversight and approval for the collection and use of routine programmatic data for monitoring and evaluation. This survey was considered programme evaluation by the U.S. Centres for Disease Control and Prevention, which is not classified as human-subjects research.

## RESULTS

### General national patient cohort

By September 30^th^ 2006, a total of 69,547 patients had accessed ART at the 102 public sector ART facilities in Malawi. Of these, 42,605 (61.3%) were female and 64,935 (93.4%) were aged 15 years or above; 12,022 patients had registered in the most recent quarter between July and September 2006; 7,692 (11%) patients had accessed ART in WHO clinical stage 1 or 2 with a CD4 count ≤250/mm^3^ , 45,386 (65%) in stage 3 in and 16,469 (24%) in stage 4. By the end of September 2006, 49,487 (71%) patients were alive and on ART. The routine national cohort analyses showed that 72% of 8,961 patients and 64% of 7,846 were alive and on ART at 6 and 12 months after enrolment, respectively. The 36 private sector facilities had registered a total of 2,781 patients by the end of September 2006. Of these, 1337 (48%) were female, 2,663 (95.6%) were aged 15 or above and 2,268 (82%) were alive and on ART. Private sector patients constituted 3.8% of the whole national cohort.

### Teachers

Information on occupation was available for 69,054 (95.5%) of 72,328 patients in the national public and private sector cohort, and 2,643 (3.7%) of these were teachers. There were 1,113 male and 1,530 (57.9%) female teachers. The mean age at ART initiation was 40.1 years (range 22–69 years) in men and 37.0 years (range 21–69 years) in women; age was unknown in 38 teachers. Seven cases had to be excluded from the analysis due to inconsistent dates of ART initiation and follow-up outcome.

The reasons for starting ART were documented in 2,607 cases (98.9%): 381 (14.5%) of these started in WHO clinical stage 1 or 2 with a CD4 count ≤250/mm^3^, 1,505 (57.1%) in stage 3 and 721 (27.4%) in stage 4. At registration, 513 (19.5%) had a history of active or previous pulmonary tuberculosis within the last 2 years, 118 (4.5%) had extra-pulmonary tuberculosis and 183 (6.9%) had Kaposi's sarcoma.

**Figure 1:**
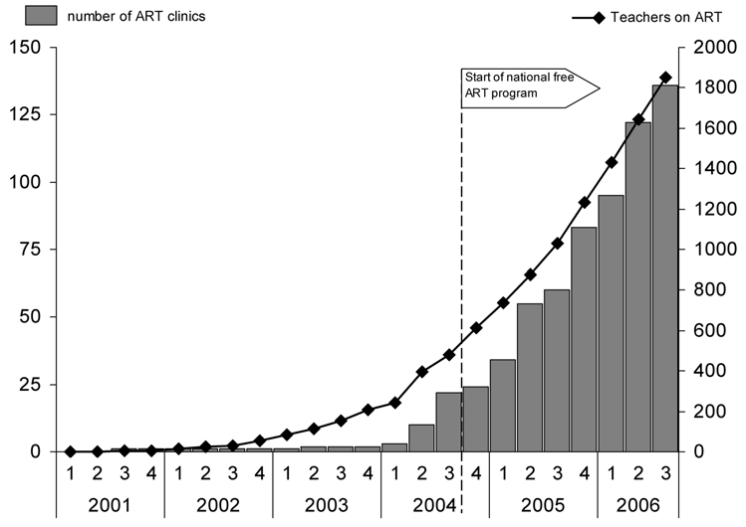
Number of ART clinics in Malawi and number of teachers on ART by the end of the respective quarter.

**Figure 1** shows the number of ART clinics in Malawi by calendar period and the number of teachers on ART at the end of each period. The first teachers in this cohort had accessed ART in January 2002 and a total of 451 (17% of the cohort) had initiated treatment in one of the 10 non-governmental clinics that had opened before the start of the national roll-out program in June 2004. The ART initiation rate increased from 75 teachers in the quarter before the national roll-out to a maximum of 340 in the first quarter of 2006. The enrolment rate slowed to 301 teachers, who initiated ART during the third quarter 2006. Overall 48% of teachers on ART had initiated treatment within the last 12 months, and the median observation time for the whole cohort was 8.5 months (inter-quartile range 3.1–17.3 months). The entire cohort had accumulated 2,575 person-years of observation. By 30^th^ September 2006, 1,850 (70.2% of teachers ever started) were alive and on ART at one of the 138 ART clinics in operation, 299 (11.3%) had died, 10 (0.4%) had stopped treatment, 268 (10.2%) had transferred out to another facility; 209 (7.9%) were lost to follow-up.

**Figure 2:**
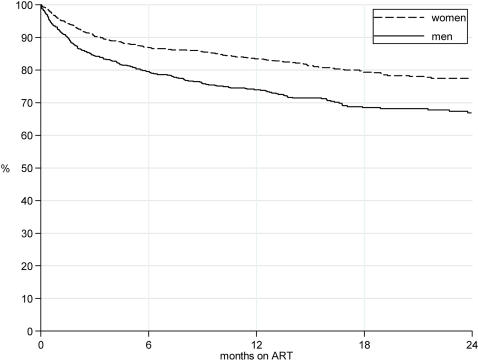
Kaplan-Meier estimates for retention of teachers in ART-program. n = 3,636; 2,575 person-years of observation.

The probability of being alive and on ART at 6, 12, 18 and 24 months was 79%, 74%, 68% and 67% for men and 86%, 83%, 79% and 76% for women, respectively (see **Figure 2**).

**Table 1** shows the distribution of sex, age and WHO Clinical Stage at ART initiation and the association of these factors with the probability of treatment drop-out (death, ART discontinuation or loss to follow-up) and mortality alone. Univariate analysis showed that men had a higher probability of treatment drop-out (HR 1.73) and drop-out rates were higher in teachers who initiated treatment under the age of 30 by comparison with the older age groups (using drop-out rates in teachers over 40 years as the reference group; HR 1.41). Drop-out rates were lower in teachers who started in WHO Clinical Stage 1 or 2 on the basis of a CD4-count of ≤250 cells/mm^3^ (HR 0.58) and higher in those who started in Stage 4 (HR 1.66). The pattern of these associations was very similar when only deaths were considered. Multivariate analysis showed that the adjusted HRs for mortality were 2.05 for men, 1.82 for the youngest age group and 1.89 for teachers who started ART in WHO Clinical stage 4.

**Table 1:** Distribution of age, sex and clinical stage of teachers at ART initiation and association with program drop-out (death, ART stop and loss to follow-up) and mortality

	n		HR drop-out (crude)		HR death (crude)		HR death (adjusted)§	
Sex								
Men	1,109	42.1%	1.73	[1.46–2.06]	2.00	[1.59–2.51]	2.05	[1.61–2.59]
Women ref	1,527	57.9%	1		1		1	
Age starting ART (years)								
21–29	179	6.8%	1.41	[1.00–1.97]	1.60	[1.05–2.45]	1.82	[1.18–2.82]
30–34	674	25.6%	1.10	[0.88–1.37]	1.04	[0.77–1.41]	1.20	[0.89–1.63]
35–39	797	30.2%	0.98	[0.79–1.22]	1.03	[0.78–1.37]	1.09	[0.82–1.44]
40–67 ref	948	36.0%	1		1		1	
Unknown	38	1.4%						
WHO Clinical Stage								
1 or 2, CD4<250	381	14.5%	0.58	[0.41–0.80]	0.39	[0.23–0.67]	0.41	[0.24–0.70]
3 ref	1,505	57.1%	1		1		1	
4	721	27.4%	1.66	[1.39–2.00]	2.01	[1.59–2.54]	1.89	[1.49–2.40]
Unknown	29	1.1%						
Total	2,636							

§ adjusted for age, sex and WHO Clinical Stage; confidence intervals in parenthesis

ref largest groups are chosen as reference category for hazard ratios

## DISCUSSION

This is the first national survey of teachers on ART examining the rate of access, retention in treatment and survival on therapy. By the end of September 2006, a total of 2,643 teachers had enrolled at one of the 138 ART clinics in Malawi. The national ART clinic data did not allow re-identification of individuals who transferred between clinics. Therefore, assuming that all teachers transferred had actually enrolled at another clinic, the 268 (10.2%) cases recorded as transferred out are likely to have been double-counted in the cohort. This reduces the actual number of individual teachers who accessed ART to approximately 2,380. Since only the ART initiation dates and not the clinic enrolment dates were known, double-counting has probably also led to an overestimation of the person-years denominator in the survival analysis. The 213 person-years of observation ending in a transfer out represented 8% (213/2,575) of the total person-year denominator and the drop-out rates and mortality are therefore likely to be approximately 8% higher than estimated. However, since observation time was probably overestimated to a similar degree in all groups, the biasing effect on the hazard ratios is probably small.

As reflected in **Figure 1**, the rate of teachers initiating ART increased considerably with the start of the free national roll-out program and retention in ART has been good, resulting in a total of 1,850 teachers alive and in ART care by the end of September 2006. According to the Department of Education Planning in the Ministry of Education, there were 43,197 primary school teachers and 10,368 secondary school teachers in the public and private education sector in 2006. Thus, 3.5% of the country's primary and secondary teachers in 2006 were alive on ART, only two years after the start of national roll-out of free ART. The majority of these teachers accessed ART in Stage 3 or 4. Given that approximately 75% of patients in these stages in Malawi are known to die within 12 months without ART [9], the roll-out of free ART is likely to have had a considerable impact on the workforce in the education sector in Malawi.

Out of the 518 ART drop-outs, 299 were deaths, 209 were due to loss to follow-up and 10 were due to discontinuing ART. Given that the patterns of association of sex, age and WHO clinical stage at treatment initiation with ART drop-out were very similar to the associations with mortality, it is likely that many of the patients who were lost to follow-up had in fact died. Adjusting for differences in the distribution of age and WHO clinical stage, male teachers had a 73% increased probability of drop-out and two-fold increase in mortality compared to women. Among the 179 (6.8%) teachers aged 12–29 years, drop-outs were 40% higher than in the oldest group aged 40–67 years; mortality was 82% higher in the youngest group. The reasons for the poorer prognosis in men and younger teachers are unclear. Some studies in Africa have identified male sex as being associated an increased risk of death [10], [11], [12], [13], although this finding is inconsistent [14], [15], [16]. This may relate to men and possibly younger people seeking care at later stages of immunodeficiency or poor treatment adherence, and the issue requires further study.

This operational study was based on data from the routine national monitoring system and has all the limitations of this type of research. Occupation was unknown for about 5% of patients, and this may have lead to an undercounting of the number of teachers who accessed ART in the country. The effects of double counting due to the inability to track transfers between clinics can be approximately quantified. Occupation is only broadly categorised in the national M&E system and it is therefore not possible to distinguish between primary, secondary and tertiary school teachers in this analysis. Due to the recent rapid expansion of ART services in Malawi, the average observation time of the national cohort is still relatively short (median 8.6 months, inter-quartile range 3.2–17.4 months) and longer term outcomes could not be reliably assessed. We are not aware of any facilities providing ART in Malawi that are not included in this study and the analysis is likely to provide near-complete information on all teachers who have accessed treatment in the country. Malawi has established a standard, national monitoring system for ART used by all public and private sector facilities. Routine validation of data is carried out during quarterly supervision of all sites, through cross-checking of master cards and ART registers, resulting in relatively high data quality.

Without doubt, the free ART programme has already mitigated the erosion of human resources in the education sector in Malawi and this effect is likely to grow more important in the future. With the progression of the national scale-up plan 2006–2010, more of the key individuals who are providing public services in rural communities will gain access to life-prolonging ART. ART may well serve as a bridge to education for all in sub-Saharan Africa [17].
